# Patient satisfaction regarding medical care for endometriosis in Germany: an exploratory cross-sectional study

**DOI:** 10.1186/s12905-026-04408-z

**Published:** 2026-03-20

**Authors:** Katharina Eisinger, Sabrina Muscharski, Miriam Schmale

**Affiliations:** Faculty Human and Health Sciences, Epidemiology, University of Bremen, Bremen, Germany

**Keywords:** Endometriosis, Patient satisfaction, PSQ-18, Medical care situation, Patient perspective

## Abstract

**Background:**

Endometriosis is a benign chronic gynecological disease in women of reproductive age. However, patient satisfaction with medical care for endometriosis in Germany remains underresearched. Therefore, this study aims to describe the medical care situation from the patient’s perspective and assess their level of satisfaction.

**Methods:**

This exploratory cross-sectional study was conducted online from July to October 2024 in Germany. A total of 350 adult participants, with confirmed, suspected, or self-assumed endometriosis completed the validated, self-administered Short-Form Patient Satisfaction Questionnaire (PSQ-18) and an extended version (PSQ-18+) with 15 endometriosis-specific items, scoring between 1 (dissatisfied) and 5 (satisfied). Descriptive analyses summarized sociodemographics, medical care and satisfaction by diagnosis status. Univariate and multiple linear regression analyses adjusted for potential confounders were used to assess the relationships between patient satisfaction and sociodemographics, disease severity and diagnosis delay. All analyses were performed using SAS 9.4.

**Results:**

Overall patient satisfaction with medical care for endometriosis in Germany was 57.87%. Satisfaction was highest for interpersonal manner (75.14%) and lowest for general aspects (41.34%), with confirmed diagnoses reporting greater satisfaction than self-assumed cases. Fewer days restricted in daily life per month (β=-0.3241, *p* = 0.0008) and fewer symptoms (β=-0.5782, *p* < 0.0001) were significantly associated with higher patient satisfaction for PSQ-18 in the univariate analyses. In the multiple analyses, fewer symptoms remained significant (β=-0.4888, *p* = 0.0001). Similar results were found for PSQ-18+.

**Conclusions:**

To improve patient satisfaction regarding the medical care for endometriosis in Germany, this study highlights patients’ perspectives, emphasizing the need to reduce diagnostic delays, improve treatment options, and support interdisciplinary, patient-centered approaches.

**Supplementary Information:**

The online version contains supplementary material available at 10.1186/s12905-026-04408-z.

## Introduction

Endometriosis is a benign chronic gynecological disease that affects girls and women in the reproductive phase of life, regardless of their ethnic origin or social status [1]. The disease is characterized by the presence of tissue similar to the endometrium outside the uterine cavity, typically in the abdominal and pelvic regions [1–4]. The etiology of the disease remains inconclusive, but theories emphasize hormonal, immunological, and genetic factors as potential causes [2, 4]. Endometriosis affects approximately 176 to 190 million (5–10%) women of reproductive age globally, with a prevalence peak occurring between the ages of 25 and 35 years [1, 5, 6].

To date, no representative data are available regarding the prevalence and incidence of diagnosed endometriosis in Germany. Estimates range widely from 0.66% to 15%, making it challenging to determine the precise prevalence [2, 3, 7, 8]. According to the Robert Koch-Institute, approximately two to four million women in Germany are affected, with around 40,000 new cases annually [8, 9].

Endometriosis is often unrecognized and difficult to diagnose due to its non-specific and complex symptoms, resulting in an average diagnostic delay of six to ten years [3, 10]. Common symptoms include premenstrual pain, dysmenorrhea, pain during urination, defecation, or sexual intercourse, as well as infertility [11].

Current management of endometriosis includes hormonal therapies [12–21], analgesics, and surgical interventions [2, 22–26], which may be combined within a multimodal treatment approach and complemented by supportive measures [27–35]. While these strategies can alleviate symptoms and improve quality of life, no curative long-term therapy is currently available, and many patients continue to experience persistent symptoms and recurrence [2, 13, 35–37]. The disease imposes substantial physical, psychological, and social burdens and is associated with reduced quality of life and considerable healthcare costs [22, 37–42].

Beyond clinical outcomes, the quality of medical care from the patients’ perspective has become increasingly important. Patient satisfaction is considered a key indicator of healthcare quality and is associated with improved health outcomes and reduced psychological burden [43–46]. This is particularly relevant in chronic conditions such as endometriosis, which typically require long-term and coordinated care. However, empirical evidence on patient satisfaction with endometriosis care in Germany remains limited, and comprehensive patient-centered analyses are scarce. In particular, there is a lack of comprehensive data capturing how affected individuals evaluate current care structures and whether these align with their needs and expectations [44].

Therefore, the primary objective of this study was to investigate patient satisfaction with the medical care of endometriosis in Germany in order to understand the needs of those affected. Secondary objectives included describing the care situation from the patients’ perspective, analyzing factors influencing the provision and utilization of medical care, and identifying potential shortcomings and areas of improvement.

## Methods

### Study design and setting

This exploratory, cross-sectional study was conducted on a national scale across Germany between July 2024 and October 2024. Data were collected with an anonymized, self-administered and structured online questionnaire via LimeSurvey [45]. A nonprobabilistic sampling approach was applied, combining convenience, snowball, and purposive sampling methods. The study was shared via the Endometriosis Association Germany e.V. and various social media platforms, with additional outreach to all endometriosis self-help groups in Germany listed on the website of the Endometriosis Association Germany e.V [47].

### Study population and eligibility criteria

As this was an exploratory cross-sectional study, no a priori sample size calculation was performed. The sample size consisted of all individuals who completed the online questionnaire during the data collection period. Participants were included if they met the following criteria: (i) aged 18 years or older, (ii) lived in Germany, (iii) had a confirmed diagnosis (ICD-10-GM: N80.- G), a suspected diagnosis (ICD-10-GM: N80.- V) [48], or a self-assumption of endometriosis, and (iv) had completed the PSQ-18 and PSQ-18 + questionnaires without missing responses for more than one item per subscale. From a total of 450 study participants, 350 individuals have met the eligibility criteria and were used for the analyses. All participants included in these analyses signed an informed consent.

### Questionnaire and study variables

A structured questionnaire was developed based on existing literature and expert input. The questionnaire consisted of three sections. The first section included medical care characteristics, such as clinical factors, symptom and health-related factors, family history of endometriosis, current pregnancy and desire to have children. The second and third sections consisted of patient satisfaction and sociodemographic factors. The questionnaire was pre-tested on 13 participants, representing all diagnostic groups to ensure comprehension and accuracy. Participants from the pretest were not included in the final analyses.

### Study instruments and operationalization

#### Diagnostic delay

Diagnostic delay timeframes were defined according to De Corte et al. [49]: The overall diagnosis time covers the complete timeframe between onset of symptoms and diagnosis. The primary diagnosis time refers to the duration from onset of symptoms to first related consultation with a doctor, whereas the clinical diagnosis time represents the duration from the first consultation with a doctor to diagnosis.

#### Patient satisfaction questionnaire short-form (PSQ-18)

Patient satisfaction was measured using the Patient Satisfaction Questionnaire Short-Form (PSQ-18), which is characterized by its robust internal consistency, reliability, and validity [50, 51]. The PSQ-18 comprises 18 items, which are divided into seven subscales and address various aspects of medical care, including general satisfaction, technical quality, interpersonal manner, communication, financial aspects, time spent with doctor, and accessibility and comfort. In addition, the PSQ-18 was expanded to encompass 15 self-assembled endometriosis-specific items and the subscales patient counseling and shared decision-making (SDM) to the PSQ-18+. Each item is evaluated on a 5-point Likert scale, ranging from 1 to 5, with higher scores indicating greater satisfaction with medical care for endometriosis. The subscale values are derived from the mean value of the assigned items [50]. To facilitate further interpretation of the satisfaction level, the mean value of each subscale was converted into a percentage using the following formula: (mean value/maximum value)×100. A list of the 15 additional items is available in Appendix 1., followed by the evaluation scheme, the allocation of individual items to the subscales in Appendices 2, 3 and 4, Additional File 1.

#### International standard classification of education (ISCED)

Socioeconomic position was approximated using the 2011 International Standard Classification of Education (ISCED-2011). It combines the highest attainment in general education with the most advanced vocational qualifications, subsequently categorizing individuals into three distinct levels: low (primary education and below, ISCED 0–2), medium (lower-secondary and upper-secondary, ISCED 3–4) and high education level (higher than upper-secondary, ISCED 5–8) [52].

#### Degree of urbanization

According to EUROSTAT’s degree of urbanization level [53], which classifies local administrative units (LAUs or municipalities), respondents were divided into categories of rural, suburban and urban areas based on their place of residence and the classification of municipalities provided by the Federal Institute for Research on Building, Urban Affairs, and Spatial Development (BBSR) [54].

### Statistical analysis

Data were analyzed using Statistical Analysis System (SAS) software version 9.4 [55]. QGIS software version 3.34.5 [56] was utilized to create the map used for the analysis of patient satisfaction by federal state (Additional File 4). Descriptive statistics were used to summarize sociodemographic characteristics, medical care and satisfaction of the study participants by diagnosis status. Continuous variables are presented as means and standard deviations (SDs), whereas categorical variables are presented as frequencies and percentages. The assumption of a normal distribution for diagnosis times and patient satisfaction was tested using visual examination of Q-Q plots and histograms, as well as analytical tests, including Shapiro-Wilk and Kolmogorov-Smirnov tests.

Univariate and multiple linear regression analyses were used to assess the relationships between various factors, including sociodemographic characteristics, disease severity and diagnosis delay, and patient satisfaction. Covariates, such as general health status, age, and ISCED, were incorporated into the multiple linear regression models to adjust for potential confounding effects. The inclusion of confounders was guided by theoretical considerations and prior evidence. Directed acyclic graphs (DAGs) were constructed for each model using DAGitty version 3.1 [57]. Subgroup analyses were conducted based on diagnosis status to explore potential differences in the determinants of patient satisfaction across these groups. All analyses were conducted with a significance level set at *p* < 0.05, and a Bonferroni correction was applied for the linear regression analyses [58].

### Handling of missing data

All available observations were included in the descriptive analyses. Participants with missing data for the primary outcomes or key confounders were excluded from the respective regression analyses. The number of data points included is reported for each model.

## Results

### Participants

Among a total of 350 participants, 78.29% had a confirmed diagnosis, 17.43% a suspected diagnosis, and 4.29% self-assumed endometriosis. The sociodemographic characteristics of the study participants are presented in Table 1. The mean age was approximately 30 years (SD = 7.7) across all participants, 31 years (SD = 7.6) for confirmed diagnosis, 27 years (SD = 7.14) for suspected diagnosis and 24 years (SD = 5.13) for self-assumed endometriosis. Most of the study population fell within the age groups of 18–27 years (42.32%) and 28–37 years (40.87%). The entire study population consisted of individuals assigned female at birth. In terms of gender identity, 96.52% identified as female, whereas 3.48% identified as non-binary. According to ISCED, nearly all participants held a middle (50%) or high (47.97%) sociodemographic position. A large majority (91.01%) of participants had statutory health insurance, with the remaining 8.99% having private health insurance. Regarding the degree of urbanization at the individual place of residence, most participants lived in cities (36.71%) or towns (37.97%), whereas 25.32% lived in rural areas.

**Table 1:** Sociodemographic characteristics (*N* = 350)

Variables	Total *n* (%)	Confirmed diagnosis *n* (%)	Suspected diagnosis *n* (%)	Self-assumption *n* (%)
N	350 (100.00)	274 (78.29)	61 (17.43)	15 (4.29)
Gender				
Female	333 (96.52)	263 (97.77)	56 (91.80)	14 (93.33)
Non-binary	12 (3.48)	6 (2.23)	5 (8.20)	1 (6.67)
Missing	5			
Age Mean; SD	30.41; 7.70	31.49; 7.60	27.18; 7.14	24.27; 5.13
Missing	5			
Age group				
18–27	146 (42.32)	95 (35.32)	39 (63.93)	12 (80.00)
28–37	141 (40.87)	124 (46.10)	15 (24.59)	2 (13.33)
38–47	46 (13.33)	39 (14.50)	6 (9.84)	1 (6.67)
48–58	12 (3.48)	11 (4.09)	1 (1.64)	0 (0.00)
Missing	5			
ISCED				
Low	7 (2.03)	5 (1.87)	2 (3.28)	0 (0.00)
Middle	172 (50.00)	127 (47.39)	36 (59.02)	9 (60.00)
High	165 (47.97)	136 (50.75)	23 (37.70)	6 (40.00)
Missing	6			
Health insurance				
Statutory	314 (91.01)	244 (90.71)	56 (91.80)	14 (93.33)
Private	31 (8.99)	25 (9.29)	5 (8.20)	1 (6.67)
Missing	5			
Degree of urbanization				
City	116 (36.71)	85 (33.86)	25 (47.17)	6 (50.00)
Town	120 (37.97)	98 (39.04)	20 (37.74)	2 (16.67)
Rural area	80 (25.32)	68 (27.09)	8 (15.09)	4 (33.33)
Missing	34			

Percentages are calculated from valid (non-missing) values, while the total N (*N* = 350) represents the complete sample

### Clinical characteristics

An overview of the health and symptom-related characteristics of the study participants is presented in Table 2. For those with self-assumed endometriosis, no information on diagnostic methods, incidental findings, receipt of a doctor’s letter and surgical removal of endometriosis was asked in the questionnaire.

**Table 2:** Symptom and health-related characteristics (*N* = 350)

Variables	Total *n* (%)	Confirmed diagnosis *n* (%)	Suspected diagnosis *n* (%)	Self-assumption *n* (%)
N	350 (100.00)	274 (78.29)	61 (17.43)	15 (4.29)
Number of initial symptoms Mean; SD*	12.79; 7.11	12.90; 7.07	11.85; 7.49	14.64; 6.20
Missing	5			
Number of symptoms within the past 6 months* Mean; SD	13.36; 7.07	13.59; 7.04	12.15; 7.20	14.20; 6.93
Missing	2			
Number of symptomatic days per month* Mean; SD	14.05; 9.61	14.93; 9.74	11.09; 8.74	9.87; 7.36
Missing	11			
Number of days restricted in daily life per month* Mean; SD	10.15; 8.98	10.98; 9.33	7.33; 6.92	6.57; 7.07
Missing	13			
Number of days on sick leave per month * Mean; SD	3.52; 6.32	3.80; 6.58	2.88; 5.67	0.92; 0.95
Missing	19			

Percentages are calculated from valid (non-missing) values, while the total N (*N* = 350) represents the complete sample

*Due to filter questions, not all participants received the complete questionnaire

Participants reported an average of 12.79 initial symptoms (SD = 7.11) and 13.36 symptoms within the past six months (SD = 7.07), as well as 14 symptomatic days per month (SD = 9.61), including 10 days of being restricted in their daily life (SD = 8.98) and 4 days on sick leave (SD = 6.32).

The average overall diagnosis time from the onset of symptoms to a confirmed diagnosis of endometriosis was 9.03 years (SD = 6.60), 7.53 years (SD = 5.87) for a suspected diagnosis. The mean primary diagnosis time was 3.33 years (SD = 4.58) for all participants with an average age of 19 years (SD = 6.74) at the onset of symptoms. The average clinical diagnosis time was 6.14 years (SD = 6.18) for a confirmed diagnosis and 3.09 years (SD = 5.02) for a suspected diagnosis.

### Patient satisfaction

Overall satisfaction with medical care for endometriosis in Germany was 57.87%, with general aspects showing the lowest satisfaction (41.34%) and interpersonal manner the highest (75.14%) (Supplementary Table 3, Additional File 2). Table 3 presents the satisfaction level of each subscale, which was divided into three diagnostic groups: 274 patients (78.29%) with a confirmed diagnosis, 61 (17.43%) with a suspected diagnosis, and 15 (4.29%) with self-assumed endometriosis.

**Table 3:** Satisfaction level of each domain of the PSQ18 by diagnosis status (*N* = 350)

Domain	Confirmed diagnosis *n* = 274		Suspected diagnosis *n* = 61		Self-assumption *n* = 15	
	Mean (SD)	Satisfaction in %	Mean (SD)	Satisfaction in %	Mean (SD)	Satisfaction in %
General Satisfaction	2.04 (0.94)	40.73	2.25 (0.91)	45.08	1.87 (0.74)	37.33
Technical Quality	3.25 (1.04)	65.05	3.05 (1.01)	60.98	2.70 (0.80)	54.00
Interpersonal Manner	3.82 (1.02)	76.39	3.61 (1.04)	72.13	3.23 (1.07)	64.67
Communication	3.22 (1.13)	64.38	3.09 (1.07)	61.80	2.60 (0.99)	52.00
Financial Aspects	2.36 (1.11)	47.23	2.91 (1.00)	58.20	3.00 (0.80)	60.00
Time Spent with Doctor	3.11 (1.17)	62.23	2.88 (1.20)	57.54	2.53 (1.01)	50.67
Accessibility and Convenience	2.54 (0.93)	50.77	2.57 (0.86)	51.48	2.52 (0.73)	50.33
Overall Satisfaction	2.91 (0.80)	58.11	2.91 (0.80)	58.17	2.64 (0.64)	52.71

Among the diagnostic groups, participants with a confirmed diagnosis reported the highest satisfaction levels in terms of technical quality (65.05%), interpersonal manner (76.39%), and communication (64.38%). Satisfaction with the time spent with a doctor was also highest in this group (62.23%). Patients with a suspected diagnosis had slightly lower satisfaction levels across most subscales, while overall satisfaction (58.17%) was comparable to that of the confirmed diagnosis group. Compared with the other diagnosis groups, general satisfaction (45.08%), as well as accessibility and convenience (51.48%), were greater for individuals with a suspected diagnosis. Patients with self-assumed endometriosis reported the lowest satisfaction levels, particularly in terms of general satisfaction (37.33%), technical quality (54%), and communication (52%). However, satisfaction with financial aspects in this group was 60%, which was higher than that in the groups of participants who received a diagnosis from a physician. Overall, differences in patient satisfaction were observed depending on the diagnostic group, with patients with a confirmed diagnosis generally reporting higher satisfaction levels.

In the extended version (PSQ-18+), which incorporates endometriosis-specific questions and the two additional subscales patient counseling and SDM, the satisfaction rates remained very similar (Table 4). For confirmed diagnosis, the satisfaction rate for the SDM subscale was the highest, reaching 65.43%. For the domains of accessibility and convenience, patient counseling, and overall satisfaction, the values for patients with confirmed and suspected diagnoses were nearly identical, at approximately 55%, 46% and 59%, respectively. Individuals with self-assumed endometriosis exhibited lower satisfaction levels across almost all subscales, especially in the areas of general satisfaction (37.33%) and patient counseling (32.53%). In terms of financial aspects, with a satisfaction rate of 60%, and accessibility and convenience, with a satisfaction rate of 58.48%, the values were higher compared to those observed among participants diagnosed by a physician. The values of the subscales of general satisfaction, financial aspects, and time spent with doctor were identical between the PSQ-18 and the PSQ-18+.

**Table 4:** Satisfaction level of each domain of the PSQ18 + by type of diagnosis (*N* = 350)

Domain	Confirmed diagnosis *n* = 274		Suspected diagnosis *n* = 61		Self-assumption *n* = 15	
	Mean (SD)	Satisfaction in %	Mean (SD)	Satisfaction in %	Mean (SD)	Satisfaction in %
General Satisfaction	2.04 (0.94)	40.73	2.25 (0.91)	45.08	1.87 (0.74)	37.33
Technical Quality	3.28 (0.94)	65.62	3.12 (0.91)	62.49	2.91 (0.73)	58.13
Interpersonal Manner	3.78 (0.87)	75.60	3.61 (0.86)	72.13	3.53 (0.85)	70.67
Communication	3.66 (0.80)	73.25	3.52 (0.80)	70.49	3.28 (0.74)	65.67
Financial Aspects	2.36 (1.11)	47.23	2.91 (1.00)	58.20	3.00 (0.80)	60.00
Time Spent with Doctor	3.11 (1.17)	62.23	2.88 (1.20)	57.54	2.53 (1.01)	50.67
Accessibility and Convenience	2.27 (0.81)	55.79	2.77 (0.75)	55.32	2.92 (0.67)	58.48
Patient Counseling	2.30 (1.07)	46.06	2.31 (1.04)	46.30	1.63 (0.55)	32.53
Shared Decision-Making (SDM)	3.27 (1.09)	65.43	3.02 (1.05)	60.33	2.60 (1.10)	52.00
Overall Satisfaction	2.96 (0.74)	59.10	2.93 (0.73)	58.65	2.70 (0.57)	53.94

### Analysis of patient satisfaction

In the univariate linear regression for sociodemographic characteristics and patient satisfaction (Supplementary Table 4, Additional File 2), health insurance emerged as the only factor statistically significantly associated with patient satisfaction on the PSQ-18 after the Bonferroni correction was applied (PSQ-18 & PSQ-18 + *p* < 0.05/5 tests = 0.01). This finding indicates a higher level of satisfaction for those with private health insurance (β = 8.61; *p* = 0.0040). For PSQ18+, no statistically significant associations were observed between sociodemographic characteristics and patient satisfaction.

The results of the univariate linear regression for disease severity and diagnostic delay with patient satisfaction are presented in Table 5 for PSQ-18 and in Supplementary Table 5, Additional File 2 for PSQ-18+. The findings indicate that individuals with fewer symptomatic days and days restricted in daily life per month, shorter clinical and overall diagnosis times, fewer symptoms and fewer initial symptoms were more likely to be overall satisfied with the medical care of endometriosis, irrespective of whether they had a confirmed or suspected diagnosis. This finding was consistent for both PSQ-18 and PSQ-18 + and for the latter was also observed for the number of days on sick leave per month. The new alpha level, as determined by the Bonferroni correction (PSQ-18 *p* < 0.05/8 tests = 0.00625; PSQ-18 + *p* < 0.05/9 tests = 0.0056), indicates a statistically significantly association between the predictor variables of the number of days restricted in daily life per month (β = -0.3241, *p* = 0.0008) as well as the number of symptoms (β = -0.5782, *p* < 0.0001) and patient satisfaction for PSQ-18. For PSQ-18+, only the number of symptoms (β = -0.03, *p* < 0.0001) was found to be statistically significant. However, the assumption of homoscedasticity was violated in the PSQ-18 analysis of the number of days on sick leave per month (Breusch-Pagan test: *p* = 0.041). Consequently, the predictor was excluded from the univariate analyses after no transformation was successful. In the PSQ-18 + analysis of number of symptoms, the assumption of a normal distribution was not fulfilled; however, a square-root transformation was successfully performed and reported.

**Table 5:** Univariate and multiple linear regression analyses for disease severity and diagnostic delay (PSQ-18)

Predictors		Univariate Regression			Multiple Regression		
	*N*	Intercept	β (CI)	*p*	Intercept	β(CI)	*p*
Number of symptomatic days per month ^a, f, h, j^	325	61.6	-0.23 (-0.48; 0.02)	0.114	61.7	-0.06 (-0.35; 0.24)	0.6013
Number of days restricted in daily life per month ^a, f, h, j^	325	61.8	-0.32 (-0.59; -0.06)	0.0008	60.3	-0.17 (-0.49; 0.16)	0.1506
Number of days on sick leave per month ^a, f, h, j^	318				62.0	-0.03 (-0.47; 0.42)	0.8552
Clinical diagnosis time (confirmed) ^b, d, e, g, i, k^	235	60.6	-0.43 (-0.90; 0.05)	0.0139	62.1	-0.31 (-0.89; 0.28)	0.14
Clinical diagnosis time (suspected) ^b, d, e, g, i, k^	47	60.4	-1.15 (-2.49; 0.19)	0.0176	37.8	-0.78 (-2.19; 0.64)	0.1149
Overall diagnosis time (confirmed) ^c, d, e, g, i, k^	228	59.4	-0.15 (-0.60; 0.31)	0.3695	67.2	-0.15 (-0.71; 0.41)	0.4638
Overall diagnosis time (suspected) ^c, d, e, g, i, k^	41	61.7	-0.88 (-2.13; 0.37)	0.0489	45.3	-0.31 (-1.89; 1.26)	0.5599
Number of initial symptoms ^a, k^	309	63.1	-0,38 (-0.76; 0.00)	0.0067	62.5	-0.37 (-0.78; 0.03)	0.0160
Number of symptoms ^a, h, j^	334	66.1	-0.58 (-0.91; -0.25)	< 0.0001	64.4	-0.49 (-0.84; -0.14)	0.0001

Results of the univariate and multiple linear regressions predicting patient satisfaction for PSQ-18. Unstandardized Regression coefficients (β), and 95% confidence interval Bonferroni corrected (CI), Significance level univariate: *p* < 0.00625, Significance level multiple: *p* < 0.00556

Predictors were adjusted for the confounders: ^a^ age, ^b^ age at first consultation with a doctor, ^c^ age at onset of symptoms, ^d^ degree of urbanization, ^e^ type of health insurance, ^f^ general health status, ^g^ ISCED, ^h^ number of chronic diseases, ^i^ number of initial symptoms, ^j^ number of therapies, ^k^ prescription of the birth control pill prior to diagnosis

The results of the multiple linear regression and the confounders considered in each model are listed in Table 5 for PSQ-18 and in Supplementary Table 5, Additional File 2 for PSQ-18+. Individuals with fewer symptomatic days, days restricted in daily life and days on sick leave per month, shorter clinical and overall diagnosis times, fewer symptoms and initial symptoms were more likely to be overall satisfied with the medical care of endometriosis, irrespective of whether they had a confirmed or suspected diagnosis. Similar findings were observed for PSQ-18+, with the only difference being that a lower number of days on sick leave per month was associated with reduced patient satisfaction. The new alpha level, as determined by the Bonferroni correction (PSQ-18 & PSQ-18 + *p* < 0.05/9 tests = 0.0056), indicates a statistically significant association between the adjusted number of symptoms (β = -0.4888, *p* = 0.0001) and patient satisfaction for PSQ-18, as well as for PSQ-18+ (β = -0.0286, *p* = 0.0002). All the conducted models explained only a small proportion of the variance in patient satisfaction, except of clinical diagnosis time (R^2^ PSQ-18 = 0.468; PSQ-18 + = 0.449) and overall diagnosis time (R^2^ PSQ-18 = 0.304; PSQ-18 + = 0.343) for the suspected diagnosis.

## Discussion

The objectives of the present study were to describe and investigate the care situation from the patients’ perspective and their satisfaction with the medical care of endometriosis, to identify potential shortcomings and areas of improvement.

Overall satisfaction with medical care for endometriosis among study participants was 57.87%, which aligns with findings from studies conducted in German-speaking countries and Australia [37, 59]. In Italy, slightly higher satisfaction rates were observed in patients who underwent a systematic process providing detailed information on medical and surgical treatment options, followed by a shared decision to initiate a structured, step-by-step care protocol. Overall satisfaction among these patients was recorded at 62% [60]. This suggests that a disease management program (DMP) for endometriosis in Germany could be a valuable opportunity to improve medical care. Such a program could establish standardized care pathways, enable early diagnosis, and deliver multidisciplinary support tailored to the patients’ complex needs. Additionally, a DMP could ensure consistent, high-quality care across different regions. By addressing unmet needs and promoting SDM, it has the potential to enhance patient satisfaction with medical care [61].

The lowest satisfaction levels were recorded in the domains of general satisfaction (41.34%), patient counseling (45.52%), financial aspects (49.69%), and accessibility and convenience (50.87%), indicating clear deficits in essential aspects of patient care, particularly in areas related to access to information about the disease and treatment options, financial burden and organizational conditions. These shortcomings may negatively affect patients’ trust in the medical care system and their treatment experiences [44, 59]. Conversely, satisfaction scores in other domains exceeded 60%, with interpersonal manner achieving the highest rating at 75.14%. This highlights the pivotal role of interpersonal interaction in shaping perceptions of medical care services [37, 59].

The values in the subscales of general satisfaction, financial aspects, and time spent with doctor are identical between the PSQ-18 and the PSQ-18+, as no new items were added to the extended version. In the domains of technical quality and interpersonal manner, as well as overall satisfaction, the differences between the two versions were minimal. Notably, communication and accessibility and convenience showed higher satisfaction scores for the PSQ-18, with differences of 9 and 5% points, respectively. The communication subscale encompasses important elements such as the comprehensibility of information and the ability to communicate without language barriers, underscoring its focus on effective and inclusive interactions between patients and healthcare providers [62, 63]. Meanwhile, the accessibility and convenience subscale provides additional insights into logistical and organizational factors, including distance, transport links, cooperation among specialists, and waiting times at the practice, which play a crucial role in shaping patients’ experiences with the healthcare system [59]. These consistent results across both versions underline the robustness of the original scale while providing deeper insights through the extended subscales. Additionally, the extended version includes two valuable subscales, patient counseling and SDM, which were not accounted for in the original PSQ-18, offering further perspectives on critical aspects of patient care.

Among the examined sociodemographic characteristics, private health insurance emerged as the only statistically significant factor influencing patient satisfaction. This finding may be attributed to the enhanced access to gynecologists and other specialists for endometriosis treatment afforded to privately insured patients [64], as well as the observed reduced diagnosis times compared with statutory health insurance. The consistent association between the presence of multiple symptoms and diminished satisfaction in the linear regression analyses, both before and after adjustment for confounders, underscores the necessity for targeted management strategies to address these symptoms. This aligns with the study findings of Lukas et al. [37], who reported that taking mental health concerns seriously and supporting women in handling their pain was associated with increased patient satisfaction regarding medical support. While Evans et al. [59] observed no association between pain severity and treatment satisfaction, others have reported pain-related symptoms, dysuria, emotional well-being and the feeling of power and control as factors influencing the quality of life of endometriosis patients, which can indirectly affect the patient satisfaction [65–67].

A Bonferroni correction was applied to account for the increased risk of Type I errors due to multiple testing [58]. While this approach is recognized as an effective strategy for reducing false positives, it is acknowledged that it can be overly conservative, resulting in diminished statistical power and an elevated risk of Type II errors. This may have resulted in the masking of true associations in the present study. Consequently, while the correction serves to enhance the validity of significant results, it may concomitantly impede the discernment of meaningful effects, thereby diminishing the interpretability of the findings [68].

The relatively low explained variance in satisfaction (except for clinical and overall diagnosis time for suspected diagnosis) in the multiple analyses suggests that other factors, may also contribute to patient satisfaction but were not captured in this study or at least were not addressed in the particular models.

This study provides a comprehensive overview of the medical care situation for endometriosis in Germany, incorporating a geographically diverse sample and considering different types of diagnosis status, including self-assumed endometriosis to account for the acknowledged high number of undiagnosed cases. Another strength is the consideration of three different diagnostic timeframes, which offers a detailed view of the stages within the diagnostic process. The inclusion of standardized measurement instruments, particularly the PSQ-18, enhances the comparability of the results with those of other studies. Given the absence of fixed threshold values for interpreting the PSQ-18, converting the scores into percentages allows for improved clarity and facilitates a more effective interpretation of the data. By integrating the patients’ perspective, valuable insights into individual care experiences and their impact on patient satisfaction are provided.

Nevertheless, there are several limitations in this study that must be acknowledged. Due to the cross-sectional study design, no causal inferences can be drawn. Because of the exploratory nature of the study, no a priori sample size calculation was performed and no formal power analysis was conducted, which may limit the statistical power. The extensive length of the questionnaire, which included several highly detailed questions, contributed to elevated drop-out rates. Furthermore, the sample size was limited for certain subgroups, particularly for those with suspected diagnoses. This may have contributed to the instability of the estimates. The study findings may not be entirely representative of the broader population of endometriosis patients, as the study primarily reached individuals who were already actively engaged with the topic through support groups, the Endometriosis Association, or social media. This has potentially led to selection bias, resulting in a sample that is more informed or invested in the topic. Furthermore, the study’s demographic composition may be considered to lack in representation, as it is predominantly composed of younger individuals, those with higher socioeconomic positions, as well as individuals living in urban areas. Consequently, the external validity and generalizability of the results to the broader endometriosis population in Germany may be limited. Given that the study data are based on participants’ self-reported information, there is a potential for social desirability bias and recall bias. Moreover, the questionnaire includes questions requiring participants to recall past experiences, inherently introducing recall bias. Additionally, owing to the self-reported nature of diagnoses and medical care factors, the accuracy of the reported data cannot be confirmed.

## Conclusions

This study revealed that many women affected by endometriosis are not completely satisfied with the medical care situation in Germany. In order to enhance the quality of care for patients diagnosed with or assumed to have endometriosis, it is crucial to reduce diagnostic delays by increasing awareness and education on endometriosis and strengthening collaboration among healthcare providers. Equally important is the implementation of holistic and interdisciplinary treatment approaches that include SDM in therapy planning to better address the diverse needs and expectations of affected individuals. Therefore, further research is needed to better understand the underlying mechanisms of endometriosis and optimize treatment strategies. In this context, future studies should prioritize increasing statistical power by including a more extensive and diverse cohort of study participants that includes all types of diagnosis status across Germany while ensuring representation of different age groups, socioeconomic situations, gender identities and ethnicities.

## Supplementary Information

Supplementary Material 1.

Supplementary Material 2.

Supplementary Material 3.

Supplementary Material 4.

## Data Availability

The datasets that were generated and/or analyzed as part of the current study are available upon reasonable request from the authors.

## Abbreviations

BBSR: Federal Institute for Research on Building, Urban Affairs and Spatial Development
CI: Confidence Interval
DAG: Directed Acyclic Graph
DMP: Disease Management Program
ICD-10-GM: International Statistical Classification of Diseases and Related Health Problems, 10. Revision, German Modification
ISCED: International Standard Classification of Education
LAU: Local Administrative Unit
PSQ-18: Patient Satisfaction Questionnaire Short-Form
PSQ-18+: Patient Satisfaction Questionnaire Short-Form extended with endometriosis-specific questions
SAS: Statistical Analysis System
SD: Standard Deviation
SDM: Shared Decision-Making

## References

[1] World Health Organization (WHO). Endometriosis. 2023. https://www.who.int/news-room/fact-sheets/detail/endometriosis. Accessed 28 Jun 2024.

[2] Deutsche Gesellschaft für Gynäkologie und Geburtshilfe e.V. (DGGG). Diagnostik und Therapie der Endometriose. Leitlinienprogramm der DGGG, SGGG und OEGGG (S2k-Level, AWMF Registry No. 045/015). 2025. https://register.awmf.org/de/leitlinien/detail/015-045

[3] KohringC, Holstiege J, Heuer J, Dammertz L, Brandes I, Mechsner S, et al. Endometriose in der vertragsärztlichen Versorgung–Regionale und zeitliche Trends im Zeitraum 2012 bis 2022. Zentralinstitut für die kassenärztliche Versorgung in Deutschland. (Zi) Versorgungsatlas-Bericht Nr. 2024;24(01). 10.20364/VA-24.01.

[4] Ulrich UA. Die Darstellung der Erkrankung. In: Becherer E, Schindler AE, editors. Endometriose ganzheitlich verstehen und behandeln Ein Ratgeber. Stuttgart: Kohlhammer; 2023. pp. 19–39.

[5] Gheorghisan-Galateanu A, Gheorghiu M. Hormonal therapy in women of reproductive age with endometriosis: an update. Acta Endocrinol (Bucharest). 2019;15(2):276–81. 10.4183/aeb.2019.276 PMC6711644 31508191

[6] Zondervan K, Becker C, Koga K, Missmer S, Taylor R, Viganò P, Endometriosis. Nat Reviews Disease Primers. 2018;4(1):9. 30026507 10.1038/s41572-018-0008-5

[7] Goehring J, Drewes M, Kalder M, Kostev K. Germany endometriosis pattern changes; prevalence and therapy over 2010 and 2019 years: a retrospective cross-sectional study. Int J Fertility Steril. 2022;16(2):85–9. 10.22074/IJFS.2021.528397.1113 PMC9108293 35639651

[8] Robert Koch-Institut (RKI). Gesundheitliche Lage der Frauen in Deutschland – wichtige Fakten auf einen Blick. Gesundheitsberichterstattung des Bundes. Berlin: Robert Koch-Institut; 2023.

[9] Robert Koch-Institut (RKI). Gesundheitliche Lage der Frauen in Deutschland. Gesundheitsberichterstattung des Bundes. Berlin: Robert Koch-Institut; 2020.

[10] Hudelist G, Fritzer N, Thomas A, Niehues C, Oppelt P, Haas D, et al. Diagnostic delay for endometriosis in Austria and Germany: causes and possible consequences. Hum Reprod. 2012;27(12):3412–6. 22990516 10.1093/humrep/des316

[11] Steck T, Felberbaum RE, Küpker W, Brucker C, Finas DF. Endometriose: Entstehung, Diagnose, Verlauf und Therapie: Springer-Verlag; 2011. 10.1007/978-3-7091-0574-0.

[12] Georgiou EX, Melo P, Baker PE, Sallam HN, Arici A, Garcia-Velasco JA, et al. Long-term GnRH agonist therapy before in vitro fertilisation (IVF) for improving fertility outcomes in women with endometriosis. Cochrane Database Syst Rev. 2019;2019(11):CD013240. 31747470 10.1002/14651858.CD013240.pub2 PMC6867786

[13] Yan H, Shi J, Li X, Dai Y, Wu Y, Zhang J, et al. Oral gonadotropin-releasing hormone antagonists for treating endometriosis-associated pain: a systematic review and network meta-analysis. Fertil Steril. 2022;118(6):1102–16. 36283862 10.1016/j.fertnstert.2022.08.856

[14] Xin L, Ma Y, Ye M, Chen L, Liu F, Hou Q. Efficacy and safety of oral gonadotropin-releasing hormone antagonists in moderate-to-severe endometriosis-associated pain: a systematic review and network meta-analysis. Arch Gynecol Obstet. 2023;308(4):1047–56. 36656435 10.1007/s00404-022-06862-0 PMC10435625

[15] Reis FM, Coutinho LM, Vannuccini S, Batteux F, Chapron C, Petraglia F. Progesterone receptor ligands for the treatment of endometriosis: the mechanisms behind therapeutic success and failure. Hum Reprod Update. 2020;26(4):565–85. 32412587 10.1093/humupd/dmaa009 PMC7317284

[16] Gibbons T, Georgiou EX, Cheong YC, Wise MR. Levonorgestrel-releasing intrauterine device (LNG-IUD) for symptomatic endometriosis following surgery. Cochrane Database Syst Rev. 2021;12(12):Cd005072. 34928503 10.1002/14651858.CD005072.pub4 PMC8686684

[17] Liu Y, Gong H, Gou J, Liu X, Li Z. Dienogest as a Maintenance Treatment for Endometriosis Following Surgery: A Systematic Review and Meta-Analysis. Front Med (Lausanne). 2021;8:652505. 33898487 10.3389/fmed.2021.652505 PMC8058209

[18] Peng C, Huang Y, Zhou Y. Dydrogesterone in the treatment of endometriosis: evidence mapping and meta-analysis. Arch Gynecol Obstet. 2021;304(1):231–52. 33398505 10.1007/s00404-020-05900-z PMC8164626

[19] Muzii L, Di Tucci C, Galati G, Carbone F, Palaia I, Bogani G, et al. The Efficacy of Dienogest in Reducing Disease and Pain Recurrence After Endometriosis Surgery: a Systematic Review and Meta-Analysis. Reprod Sci. 2023;30(11):3135–43. 37217824 10.1007/s43032-023-01266-0 PMC10643411

[20] Brown J, Crawford TJ, Datta S, Prentice A. Oral contraceptives for pain associated with endometriosis. Cochrane Database Syst Rev. 2018;5(5):Cd001019. 29786828 10.1002/14651858.CD001019.pub3 PMC6494634

[21] Jensen JT, Schlaff W, Gordon K. Use of combined hormonal contraceptives for the treatment of endometriosis-related pain: a systematic review of the evidence. Fertil Steril. 2018;110(1):137–52. 29937152 10.1016/j.fertnstert.2018.03.012

[22] Horne AW, Missmer SA. Pathophysiology, diagnosis, and management of endometriosis. BMJ. 2022;379:e070750. 36375827 10.1136/bmj-2022-070750

[23] Halis G, Mechsner S, Ebert AD. The diagnosis and treatment of deep infiltrating endometriosis. Deutsches Ärzteblatt international. 2010;107(25):446–56. 20644707 10.3238/arztebl.2010.0446 PMC2905889

[24] Bafort C, Beebeejaun Y, Tomassetti C, Bosteels J, Duffy JM. Laparoscopic surgery for endometriosis. Cochrane Database Syst Rev. 2020;10(10):Cd011031. 33095458 10.1002/14651858.CD011031.pub3 PMC8428328

[25] Singh SS, Gude K, Perdeaux E, Gattrell WT, Becker CM. Surgical Outcomes in Patients With Endometriosis: A Systematic Review. J Obstet Gynaecol Can. 2020;42(7):881–8. 31718952 10.1016/j.jogc.2019.08.004

[26] Song Z, Li S, Luo M, Li H, Zhong H, Wei S. Assessing the role of robotic surgery versus laparoscopic surgery in patients with a diagnosis of endometriosis: A meta-analysis. Med (Baltim). 2023;102(50):e33104. 10.1097/MD.0000000000033104 PMC10727685 38115379

[27] Gao Q, Shen L, Jiang B, Luan YF, Lin LN, Meng FC, et al. Salvia miltiorrhiza-Containing Chinese Herbal Medicine Combined With GnRH Agonist for Postoperative Treatment of Endometriosis: A Systematic Review and meta-Analysis. Front Pharmacol. 2022;13:831850. 35250579 10.3389/fphar.2022.831850 PMC8889030

[28] Wu Y, Liu Y, Jia H, Luo C, Chen H. Treatment of endometriosis with dienogest in combination with traditional Chinese medicine: A systematic review and meta-analysis. Front Surg. 2022;9:992490. 36386543 10.3389/fsurg.2022.992490 PMC9663487

[29] Nirgianakis K, Egger K, Kalaitzopoulos DR, Lanz S, Bally L, Mueller MD. Effectiveness of Dietary Interventions in the Treatment of Endometriosis: a Systematic Review. Reprod Sci. 2022;29(1):26–42. 33761124 10.1007/s43032-020-00418-w PMC8677647

[30] Baradwan S, Gari A, Sabban H, Alshahrani MS, Khadawardi K, Bukhari IA, et al. The effect of antioxidant supplementation on dysmenorrhea and endometriosis-associated painful symptoms: a systematic review and meta-analysis of randomized clinical trials. Obstet Gynecol Sci. 2024. 10.5468/ogs.23210. 10.5468/ogs.23210 PMC10948216 38221738

[31] Zheng SH, Chen XX, Chen Y, Wu ZC, Chen XQ, Li XL. Antioxidant vitamins supplementation reduce endometriosis related pelvic pain in humans: a systematic review and meta-analysis. Reprod Biol Endocrinol. 2023;21(1):79. 37644533 10.1186/s12958-023-01126-1 PMC10464024

[32] Donatti L, Malvezzi H, Azevedo BC, Baracat EC, Podgaec S. Cognitive Behavioral Therapy in Endometriosis, Psychological Based Intervention: A Systematic Review. Rev Bras Ginecol Obstet. 2022;44(3):295–303. 35576938 10.1055/s-0042-1742406 PMC9948268

[33] Samami E, Shahhosseini Z, Khani S, Elyasi F. Pain-focused psychological interventions in women with endometriosis: A systematic review. Neuropsychopharmacol Rep. 2023;43(3):310–9. 37366616 10.1002/npr2.12348 PMC10496056

[34] Hansen S, Sverrisdóttir U, Rudnicki M. Impact of exercise on pain perception in women with endometriosis: A systematic review. Acta Obstet Gynecol Scand. 2021;100(9):1595–601. 33999412 10.1111/aogs.14169

[35] Mechsner S. Ganzheitliche Behandlung der Endometriose. Der Schmerz. 2023;37(6):437–347. 37626190 10.1007/s00482-023-00747-0

[36] Imboden S, Mueller M. Quality of life in patients with endometriosis. Gynäkologische Endokrinologie. 2018;16:76–9.

[37] Lukas I, Kohl-Schwartz A, Geraedts K, Rauchfuss M, Wölfler MM, Häberlin F, et al. Satisfaction with medical support in women with endometriosis. PLoS ONE. 2018;13(11):e0208023. 30496315 10.1371/journal.pone.0208023 PMC6264517

[38] Culley L, Law C, Hudson N, Denny E, Mitchell H, Baumgarten M, et al. The social and psychological impact of endometriosis on women’s lives: a critical narrative review. Hum Reprod Update. 2013;19(6):625–39. 23884896 10.1093/humupd/dmt027

[39] Friedl F, Riedl D, Fessler S, Wildt L, Walter M, Richter R, et al. Impact of endometriosis on quality of life, anxiety, and depression: an Austrian perspective. Arch Gynecol Obstet. 2015;292:1393–9. 26112356 10.1007/s00404-015-3789-8

[40] Agarwal SK, Foster WG, Groessl EJ. Rethinking endometriosis care: applying the chronic care model via a multidisciplinary program for the care of women with endometriosis. Int J women’s health. 2019:405–10. 10.2147/IJWH.S207373. 10.2147/IJWH.S207373 PMC6661982 31413643

[41] Simoens S, Dunselman G, Dirksen C, Hummelshoj L, Bokor A, Brandes I, et al. The burden of endometriosis: costs and quality of life of women with endometriosis and treated in referral centres. Hum Reprod. 2012;27(5):1292–9. 22422778 10.1093/humrep/des073

[42] Soliman AM, Yang H, Du EX, Kelley C, Winkel C. The direct and indirect costs associated with endometriosis: a systematic literature review. Hum Reprod. 2016;31(4):712–22. 26851604 10.1093/humrep/dev335

[43] Bertakis KD, Callahan EJ, Helms LJ, Azari R, Robbins JA, Miller J. Physician practice styles and patient outcomes: differences between family practice and general internal medicine. Med Care. 1998;36(6):879–91. 9630129 10.1097/00005650-199806000-00011

[44] Neugebauer B, Porst R. Patientenzufriedenheit: ein Literaturbericht. (ZUMA-Methodenbericht, 2001/07). Mannheim: Zentrum für Umfragen, Methoden und Analysen -ZUMA-; 2001.

[45] Denny E, Mann CH. Endometriosis and the primary care consultation. Eur J Obstet Gynecol Reproductive Biology. 2008;139(1):111–5. 10.1016/j.ejogrb.2007.10.006 18068292

[46] Perneger TV. What’s wrong with Bonferroni adjustments. BMJ. 1998;316(7139):1236–8. 10.1136/bmj.316.7139.1236 PMC1112991 9553006

[47] LimeSurvey GmbH. LimeSurvey: An open source survey tool. n.d. https://www.limesurvey.org. Accessed 28 Oct 2024.

[48] Endometriose Vereinigung Deutschland e.V. Homepage - Endometriose Vereinigung Deutschland e.V. n.d. https://www.endometriose-vereinigung.de/. Accessed 23 Jan 2025.

[49] Bundesinstitut für Arzneimittel und Medizinprodukte (BfArM). Bundesministerium für Gesundheit (BMG), Arbeitsgruppe ICD des Kuratoriums für Fragen der Klassifikation im Gesundheitswesen (KKG). ICD-10-GM Version 2024 Systematisches Verzeichnis - Internationale statistische Klassifikation der Krankheiten und verwandter Gesundheitsprobleme, 10. Revision, German Modification, Stand 05. September 2023. Köln: Bundesinstitut für Arzneimittel und Medizinprodukte; 2023.

[50] De Corte P, Klinghardt M, von Stockum S, Heinemann K. Time to Diagnose Endometriosis: Current Status, Challenges and Regional Characteristics—A. Syst Literature Rev BJOG: Int J Obstet Gynecol. 2025;132(2):118–30. 10.1111/1471-0528.17973 PMC11625652 39373298

[51] Marshall GN, Hays RD. The patient satisfaction questionnaire short-form (PSQ-18). Rand Santa Monica, CA; 1994. 23883565 10.3402/meo.v18i0.21747 PMC3722414

[52] Thayaparan AJ, Mahdi E. The Patient Satisfaction Questionnaire Short Form (PSQ-18) as an adaptable, reliable, and validated tool for use in various settings. Med Educ Online. 2013;18(1):21747. 10.3402/meo.v18i0.21747. 10.3402/meo.v18i0.21747 PMC3722414 23883565

[53] UNESCO Institute for Statistics (UIS). International Standard Classification of Education: ISCED 2011. Montreal (QC). UNESCO Institute for Statistics; 2012.

[54] Eurostat. Degree of Urbanisation: Methodology. n.d. https://ec.europa.eu/eurostat/web/degree-of-urbanisation/methodology. Accessed 23 Mar 2025.

[55] Bundesinstitut für Bau-, Stadt- und Raumforschung (BBSR). Raumgliederungssystem des Bundesinstituts für Bau-, Stadt- und Raumforschung (BBSR) zum Gebietsstand 31.12.2022. 2024. https://www.bbsr.bund.de/BBSR/DE/forschung/raumbeobachtung/Raumabgrenzungen/downloads/raumgliederungen-referenzen-2022.xlsx?__blob=publicationFile&v=8. Accessed 23 Mar 2025.

[56] SAS Institute. SAS 9.4 [software]. Cary, NC, USA: SAS Institute; n.d.

[57] QGIS Development Team. QGIS Geographic Information System [software]. Version 3.34.5. Grüt (Gossau ZH), Switzerland: Open Source Geospatial Foundation Project; n.d. https://qgis.org/.

[58] Textor J, van der Zander B, Gilthorpe MK, Liśkiewicz M, Ellison GTH. DAGitty — draw and analyze causal diagrams [software]. Version 3.1. Nijmegen, Netherlands: Radboud University; n.d. https://www.dagitty.net/.

[59] Sedgwick P. Multiple significance tests: the Bonferroni correction. BMJ. 2012;344. 10.1136/bmj.e509. 34396651 10.1111/imj.15494

[60] Evans S, Villegas V, Dowding C, Druitt M, O’Hara R, Mikocka-Walus A. Treatment use and satisfaction in Australian women with endometriosis: a mixed‐methods study. Intern Med J. 2022;52(12):2096–106. 10.1111/imj.15494. 34396651 10.1111/imj.15494

[61] Vercellini P, Donati A, Ottolini F, Frassineti A, Fiorini J, Nebuloni V, et al. A stepped-care approach to symptomatic endometriosis management: a participatory research initiative. Fertil Steril. 2018;109(6):1086–96. 10.1016/j.fertnstert.2018.01.037 29871796

[62] Kassenärztliche Bundesvereinigung (KBV). Disease-Management-Programme. 2024. https://www.kbv.de/html/dmp.php. Accessed 10 Mar 2025.

[63] Cox H, Ski CF, Wood R, Sheahan M. Endometriosis, an unknown entity: the consumer’s perspective. Int J consumer Stud. 2003;27(3):200–9. 37947536 10.3390/ijerph20216978 PMC10649906

[64] Cofini V, Muselli M, Limoncin E, Lolli C, Pelaccia E, Guido M, et al. The Perception of the Quality of Professional Healthcare Assistance for the Management of Endometriosis: Findings from a National Survey in Italy. Int J Environ Res Public Health. 2023;20(21):6978. 10.3390/ijerph20216978 PMC10649906 37947536

[65] Fourquet J, Zavala DE, Missmer S, Bracero N, Romaguera J, Flores I. Disparities in healthcare services in women with endometriosis with public vs private health insurance. Am J Obstet Gynecol. 2019;221(6):623. e1-. e11. 10.1016/j.ajog.2019.06.020 31226295

[66] Arboleda AM. Reprint of: Satisfaction with life and perception of healthcare services. Int J Hospitality Manage. 2023;112:103516. 38374495 10.1111/nhs.13100

[67] Wu YH, Lu YY, Liu KF. Factors influencing health-related quality of life in women with endometriosis: A cross‐sectional study. Nurs Health Sci. 2024;26(1):e13100. 38374495 10.1111/nhs.13100

[68] Kupec T, Kennes LN, Senger R, Meyer-Wilmes P, Najjari L, Stickeler E, et al. The Multifactorial Burden of Endometriosis: Predictors of Quality of Life. J Clin Med. 2025;14(2):323. 39860327 10.3390/jcm14020323 PMC11765614

