# Supplementary material for: Patient satisfaction regarding medical care for endometriosis in Germany: an exploratory cross-sectional study
Source: BMC Womens Health. 2026 Mar 20;26:197. doi: 10.1186/s12905-026-04408-z (PMC13063557; doi:10.1186/s12905-026-04408-z)
Supplement: Supplementary file 1 — Supplementary Material 1. [file 12905_2026_4408_MOESM1_ESM.docx]

**Further explanations on methods**

It is important to note that some PSQ-18 items are formulated in such a manner that agreement serves as a reflection of satisfaction with medical care, whereas other items are formulated to serve as a reflection of dissatisfaction with medical care. In order to standardize the evaluation process, it is imperative that all items are evaluated in such a way that agreement is the primary indicator of satisfaction with medical care. The evaluation scheme according to Marshall and Hays (1994) is illustrated in the ensuing Appendix 2.

**Appendix 1** Additional Questions of the of PSQ-18+

| Item Number | Questions |
| --- | --- |
| E1 | I am satisfied with the clarity of the information I receive from my doctor. |
| E2 | I am satisfied with the extent to which I am involved in decision-making. |
| E3 | I am satisfied with the collaboration between the doctors treating me for my endometriosis (e.g., communication between doctors, referral of doctor’s letter). |
| E4 | I am satisfied with the professional competence of the non-medical practice staff. |
| E5 | I am satisfied with how the non-medical practice staff treats me. |
| E6 | I am satisfied with the waiting time at the practice. |
| E7 | My doctor is easily accessible for me (e.g., distance, transportation, accessibility, barrier-free access). |
| E8 | My doctor and I can communicate without any language barriers (regarding the national language). |
| E9 | My doctor has provided me with detailed information about endometriosis (e.g., etiology, disease progression, symptoms). |
| E10 | My doctor has provided me with detailed information about different treatment options for endometriosis. |
| E11 | My doctor has discussed the benefits of each treatment option with me. |
| E12 | My doctor has discussed the disadvantages of each treatment option with me (e.g., side effects). |
| E13 | My doctor has coordinated the therapy/therapies with me. |
| E14 | My doctor considers my current needs in the treatment (e.g., desire to have children, hormone-free therapy). |
| E15 | My doctor has informed me about psychological support options (e.g., therapy sessions) for coping with endometriosis |

**Appendix 2** Scoring of the PSQ-18 items

| Item Numbers | Original Response Value | Scored Value |
| --- | --- | --- |
| 1, 2, 3, 5, 6, 8, 11, 15, 18 | 1 ----------------->  2 ----------------->  3 ----------------->  4 ----------------->  5 -----------------> | 5  4  3  2  1 |
| 4, 7, 9, 10, 12, 13, 14, 16, 17 | 1 ----------------->  2 ----------------->  3 ----------------->  4 ----------------->  5 -----------------> | 1  2  3  4  5 |

Appendices 3 and 4 illustrate the allocation of individual items to the subscales of the PSQ-18 and PSQ-18+, respectively.

**Appendix 3** Allocation of the items to the subscales of the PSQ-18 and creation of scale scores

| Subscale | Average of these items |
| --- | --- |
| General Satisfaction | 3, 17 |
| Technical Quality | 2, 4, 6, 14 |
| Interpersonal Manner | 10, 11 |
| Communication | 1, 13 |
| Financial Aspects | 5, 7 |
| Time Spent with Doctor | 12, 15 |
| Accessibility and Convenience | 8, 9, 16, 18 |

**Appendix 4** Allocation of the items to the subscales of the PSQ-18+ and creation of scale scores

| Subscale | Average of these items |
| --- | --- |
| General Satisfaction | P3, P17 |
| Technical Quality | P2, P4, P6, P14, E4 |
| Interpersonal Manner | P10, P11, E5 |
| Communication | P1, P13, E1, E8 |
| Financial Aspects | P5, P7 |
| Time Spent with Doctor | P12, P15 |
| Accessibility and Convenience | P8, P9, P16, P18, E3, E6, E7 |
| Patient Counseling | E9, E10, E11, E12, E15 |
| Shared Decision-Making (SDM) | E2, E13, E14 |

P: Item from the PSQ-18, E: Item from the extension.
