# Supplementary material for: Patient satisfaction regarding medical care for endometriosis in Germany: an exploratory cross-sectional study
Source: BMC Womens Health. 2026 Mar 20;26:197. doi: 10.1186/s12905-026-04408-z (PMC13063557; doi:10.1186/s12905-026-04408-z)
Supplement: Supplementary file 2 — Supplementary Material 2. [file 12905_2026_4408_MOESM2_ESM.docx]

Patient satisfaction regarding medical care for endometriosis in Germany: An exploratory cross-sectional study

**Dear Participants,**

this is a study on satisfaction with the medical care for endometriosis in Germany. It includes questions covering first symptoms, diagnosis and treatment. This survey is aimed at people who have been diagnosed with endometriosis or suspect they may have endometriosis, and who are at least 18 years old.

We are four Master’s students in Epidemiology at the University of Bremen and with our study, we want to gain new insights and approaches for future research on this topic. We kindly ask you to share your experiences and opinions about your medical care openly and honestly. Your data will be collected anonymously, treated confidentially, and cannot be traced back to you.

Completing the questionnaire will take approximately 20 minutes. If you need to interrupt the survey, you may return later to finish it. For better usability, we recommend completing the survey in desktop view. If you have a doctor’s letter available, you may use them to help answer certain questions, though this is not mandatory for answering the questions.

Thank you very much in advance for your time and willingness to participate. If you have questions or comments, feel free to contact us at: endometriose-studie@gmx.de.

**Best regards,**
Katharina Eisinger, Sabrina Muscharski & Miriam Schmale

**[G00Q01] Are you personally affected by endometriosis?**

Please choose one of the following answers:

- Yes (confirmed diagnosis, suspected diagnosis, own suspicion)
- No

# Questions about your endometriosis

**[G01Q01] Think back to the beginning of your endometriosis. Do you remember which symptoms made you or someone else first suspect endometriosis?**

Please choose one of the following answers:

- Yes
- No, I no longer remember which symptoms I had initially
- I had no specific symptoms

**[G01Q02] What were the symptoms that made you or someone else first suspect endometriosis?** (Only displayed if “yes” in [G01Q01])

**[G01Q03] Symptoms related to the reproductive organs** (Only displayed if “yes” in [G01Q01])

Select all answers that apply:

- Menstrual abdominal pain
- Non-menstrual abdominal pain
- Abdominal pain during ovulation
- Irregular cycle
- Heavy menstrual bleeding
- Prolonged menstrual bleeding
- Breast tenderness
- Absence of menstruation
- Infertility

**[G01Q04] General symptoms** (Only displayed if “yes” in [G01Q01])

Select all answers that apply:

- Exhaustion
- Fatigue
- Sleep disorders
- Night sweats
- Mood swings
- Dizziness
- Nausea
- Vomiting
- Flatulence
- Diarrhea
- Constipation
- Urge to urinate

**[G01Q05] Pain** (Only displayed if “yes” in [G01Q01])

Select all answers that apply:

- Headaches
- Migraine
- Shoulder pain
- Chest pain
- Back pain
- Pain radiating into the legs
- Pain during intercourse
- Pain after intercourse
- Pain when urinating
- Pain when defecating

**[G01Q06] Did you have other or additional initial symptoms not listed above?** (Only displayed if “yes” in [G01Q01])

Please enter your answer here:

Open text

**[G01Q07] When did these symptoms first appear?** (Only displayed if “yes” in [G01Q01])

Enter year ≤ 2024

**[G01Q08] Did you ever visit a doctor because of these symptoms linked to endometriosis?**

Please choose one of the following answers:

- Yes
- No

**[G01Q09] When did you first visit a doctor because of these symptoms linked to endometriosis?** (Only displayed if “yes” in [G01Q08])

Enter year ≤ 2024

# Questions on diagnosis of endometriosis

**[G02Q01] Do you have a confirmed or a suspected diagnosis of endometriosis?**

A suspected diagnosis means that your doctor suspects endometriosis, but there is not yet definitive proof. In contrast, a confirmed diagnosis means that various medical tests have verified the presence of endometriosis. Both types of diagnoses should be documented in your doctor’s letter in the form of the “**ICD-10 code**”: **Endometriosis [N80.0] to [N80.9].**
If the ICD-10 code ends with the letter **“G”**, it is a **confirmed diagnosis.**
If the ICD-10 code ends with the letter **“V”**, it is a **suspected diagnosis.**

Please choose one of the following answers:

- Confirmed diagnosis
- Suspected diagnosis
- Neither, but I personally suspect it

**[G02Q02] When did you receive your confirmed diagnosis?** (Only displayed if “confirmed diagnosis” in [G02Q01])

Enter year ≤ 2024

**[G02Q03] When did you receive your suspected diagnosis?** (Only displayed if “confirmed diagnosis” in [G02Q01])

Enter year ≤ 2024

**[G02Q04] Did you receive a doctor’s letter from any treating practice or clinic?** (Only displayed if “confirmed diagnosis” or “suspected diagnosis” in [G02Q01])

Please choose one of the following answers:

- Yes
- No

**[G02Q05] Was your endometriosis discovered incidentally?** (Only displayed if ‘confirmed diagnosis’ or ‘suspected diagnosis’ in [G02Q01])

An incidental finding means that the endometriosis was discovered during a medical procedure that was not performed for the purpose of diagnosing endometriosis, for example during an examination or surgery conducted for another reason.

Please choose one of the following answers:

- Yes
- No
- Unknown

**[G02Q06] Which procedures were performed to diagnose your endometriosis?** (Only displayed if “confirmed diagnosis” or “suspected diagnosis” in [G02Q01])

Select all answers that apply:

- Consultation with a doctor
- Physical examination (e.g. palpation)
- Sonography (ultrasound)
- Magnetic resonance imaging (MRI)
- Computed tomography (CT), X-ray
- Seen by doctor during surgery
- Laboratory examination of tissue collected during surgery
- Unknown
- Other (open text)

# Questions on classification of endometriosis

**[G03Q01] How was your endometriosis classified?**

There are various ways to determine the stage of endometriosis, including the AFS/rASRM stage as well as the ENZIAN and #ENZIAN classifications. You should also find information about this in your doctor’s letter.

Select all answers that apply:

- AFS stage
- rASRM stage
- ENZIAN classification
- #ENZIAN classification
- Unknown

**[G03Q02] Which AFS/rASRM stage applies to you?** (Only displayed if “AFS stage” or “rASRM stage” in [G03Q01])

Please choose one of the following answers:

- Stage I
- Stage II
- Stage III
- Stage IV
- unknown

**[G03Q03] What is your ENZIAN/#ENZIAN classification?** (Only displayed if “ENZIAN classification” or “#ENZIAN classification” in [G03Q01])

Please select the answer that applies to each item:

|  | 1 | 2 | 3 | unknown | not stated |
| --- | --- | --- | --- | --- | --- |
| **P** | ○ | ○ | ○ | ○ | ○ |
| **O (left)** | ○ | ○ | ○ | ○ | ○ |
| **O (right)** | ○ | ○ | ○ | ○ | ○ |
| **T (left)** | ○ | ○ | ○ | ○ | ○ |
| **T (right)** | ○ | ○ | ○ | ○ | ○ |
| **A** | ○ | ○ | ○ | ○ | ○ |
| **B** | ○ | ○ | ○ | ○ | ○ |
| **C** | ○ | ○ | ○ | ○ | ○ |

**[G03Q04] If you have an F-classification, please enter it here.** (Only displayed if “ENZIAN classification” or “#ENZIAN classification” in [G03Q01])

Select all answers that apply:

- FA

- FB

- FI

- FU

- Other (open text)

# Questions on symptoms of endometriosis

**[G04Q01] Have you had symptoms within the last 6 months that you associate with endometriosis?**

Please choose one of the following answers:

- Yes
- No

**[G04Q02] Which symptoms have you had in the last 6 months?** (Only displayed if “yes” in [G04Q01])

**[G04Q03] Symptoms related to the reproductive organs** (Only displayed if “yes” in [G04Q01])

Select all answers that apply:

- Menstrual abdominal pain
- Non-menstrual abdominal pain
- Abdominal pain during ovulation
- Irregular cycle
- Heavy menstrual bleeding
- Prolonged menstrual bleeding
- Breast tenderness
- Absence of menstruation
- Infertility

**[G04Q04] General symptoms** (Only displayed if “yes” in [G04Q01])

Select all answers that apply:

- Exhaustion
- Fatigue
- Sleep disorders
- Night sweats
- Mood swings
- Dizziness
- Nausea
- Vomiting
- Flatulence
- Diarrhea
- Constipation
- Urge to urinate

**[G04Q05] Pain** (Only displayed if “yes” in [G04Q01])

Select all answers that apply:

- Headaches
- Migraine
- Shoulder pain
- Chest pain
- Back pain
- Pain radiating into the legs
- Pain during intercourse
- Pain after intercourse
- Pain when urinating
- Pain when defecating

**[G04Q06] Did you have other or additional symptoms not listed above in the last 6 months?** (Only displayed if “yes” in [G04Q01])

Please enter your answer here:

Open text

**[G04Q07] On average, how many days per month does at least one of these symptoms occur?** (Only displayed if “yes” in [G04Q01])

Refer to the last 6 months:

On approximately (open text) days per month.

**[G04Q08] On average, how many days per month are you limited in everyday life due to your endometriosis?** Only displayed if “yes” in [G04Q01])

Refer to the last 6 months:

On approximately (open text) days per month.

**[G04Q09] On average, how many days per month are you unable to work or on sick leave due to endometriosis?** (Only displayed if “yes” in [G04Q01])

Refer to the last 6 months:

On approximately (open text) days per month.

# Questions on treatment of endometriosis

**[G05Q01] Where are you being treated for endometriosis?**

Select all answers that apply:

- General practitioner
- Gynecologist
- Endometriosis center
- Psychiatrist
- Gastroenterologist
- Endocrinologist
- Reproductive physician
- Nutritionist
- Naturopath
- Other (open text)

**[G05Q02] How is your endometriosis currently treated?**

Please indicate in the open text field in which year you started to use the therapy.

Select all answers that apply and write a comment:

- Hormonal therapy (+ open text)
- Medication-based pain therapy (+ open text)
- Diet change (+ open text)
- Exercise and physical activity (+ open text)
- Physiotherapy (+ open text)
- Complementary therapies (+ open text)
- Psychotherapy (+ open text)
- Other (+ open text)

**[G05Q03] Do you experience complaints or side effects from your treatment?**

Please enter your answer here:

Open text

**[G05Q04] Which therapies have you previously used and then stopped?**

Please indicate in the open text field when you received the respective therapy (e.g. 2015-2018).

Select all answers that apply and write a comment:

- Hormonal therapy (+ open text)
- Medication-based pain therapy (+ open text)
- Diet change (+ open text)
- Exercise and physical activity (+ open text)
- Physiotherapy (+ open text)
- Other complementary therapies (+ open text)
- Psychotherapy (+ open text)
- No therapies have been discontinued yet (+ open text)
- Other (+ open text)

**[G05Q05] Why were these therapies discontinued?** (Only displayed if not “No therapies have been discontinued yet” in [G05Q04])

Please enter your answer here:

Open text

# Additional questions on treatment

**[G06Q01] Were you prescribed the birth control pill for your endometriosis?**

Please choose one of the following answers:

- Yes, the birth control pill was prescribed to me specifically

- No, I was already taking the birth control pill

- No, the birth control pill was prescribed to me for another reason

- No, the birth control pill was not prescribed to me

**[G06Q02] Was your endometriosis surgically removed?** (Only displayed if “confirmed diagnosis” or “suspected diagnosis” in [G02Q01])

- Yes

- No, but I will have surgery soon

- No

**[G06Q03] Have you had a hysterectomy?**

Please choose one of the following answers:

- Yes, due to endometriosis

- Yes, for other reasons

- No

- I was born without a uterus

**[G06Q04] Are you currently pregnant?** (Only displayed if “no” in [G06Q03])

Please choose one of the following answers:

- Yes

- No

**[G06Q05] Do you generally wish to have children?** (Only displayed if "no” in [G06Q03] and “no” in [G06Q04])

Please choose one of the following answers:

- Yes, I currently want to have children

- Yes, I can imagine having children at some point

- No

**[G06Q06] Do you have any other chronic diseases?**

Please choose one of the following answers:

- No

- Yes (open text)

**[G06Q07] Has or had another family member endometriosis?**

Please choose one of the following answers:

- Yes

- No

- Unknown

# Questions on satisfaction with medical care

In the following, we ask you a few questions about your satisfaction with the medical care you receive for your endometriosis. Please refer to the doctor who treats your endometriosis (usually your gynecologist).

**[G07Q01] How much do you agree with the statements?**

Please indicate how much you agree with the following statements:

|  | Strongly agree | Agree | Uncertain | Disagree | Strongly disagree |
| --- | --- | --- | --- | --- | --- |
| Doctors are good about explaining the reason for medical tests. | ○ | ○ | ○ | ○ | ○ |
| I think my doctor’s office has everything needed to provide complete medical care. | ○ | ○ | ○ | ○ | ○ |
| The medical care I have been receiving is just about perfect. | ○ | ○ | ○ | ○ | ○ |
| Sometimes doctors make me wonder if their diagnosis is correct. | ○ | ○ | ○ | ○ | ○ |
| I feel confident that I can get the medical care I need without being set back financially. | ○ | ○ | ○ | ○ | ○ |
| When I go for medical care, they are careful to check everything when treating and examining me. | ○ | ○ | ○ | ○ | ○ |
| I have to pay for more of my medical care than I can afford. | ○ | ○ | ○ | ○ | ○ |
| I have easy access to the medical specialists I need. | ○ | ○ | ○ | ○ | ○ |
| Where I get medical care, people have to wait too long for emergency treatment. | ○ | ○ | ○ | ○ | ○ |
| Doctors act too businesslike and impersonal toward me. | ○ | ○ | ○ | ○ | ○ |
| My doctors treat me in a very friendly and courteous manner. | ○ | ○ | ○ | ○ | ○ |
| Those who provide my medical care sometimes hurry too much when they treat me. | ○ | ○ | ○ | ○ | ○ |
| Doctors sometimes ignore what I tell them. | ○ | ○ | ○ | ○ | ○ |
| I have some doubts about the ability of the doctors who treat me. | ○ | ○ | ○ | ○ | ○ |
| Doctors usually spend plenty of time with me. | ○ | ○ | ○ | ○ | ○ |
| I find it hard to get an appointment for medical care right away. | ○ | ○ | ○ | ○ | ○ |
| I am dissatisfied with some things about the medical care I receive. | ○ | ○ | ○ | ○ | ○ |
| I am able to get medical care whenever I need it. | ○ | ○ | ○ | ○ | ○ |

# Additional questions on satisfaction

**[G08Q01] How much do you agree with the statements?**

Please indicate how much you agree with the following statements:

|  | Strongly agree | | | Agree | Uncertain | | Disagree | Strongly disagree |
| --- | --- | --- | --- | --- | --- | --- | --- | --- |
| I am satisfied with the clarity of the information I receive from my doctor. | ○ | | | ○ | ○ | | ○ | ○ |
| I am satisfied with the extent to which I am involved in decision-making. | ○ | | | ○ | ○ | | ○ | ○ |
| I am satisfied with the collaboration between the doctors treating me for my endometriosis (e.g., communication between doctors, referral of doctor’s letter). | ○ | | | ○ | ○ | | ○ | ○ |
| I am satisfied with the professional competence of the non-medical practice staff. | ○ | | | ○ | ○ | | ○ | ○ |
| I am satisfied with how the non-medical practice staff treat me. | ○ | | | ○ | ○ | | ○ | ○ |
| I am satisfied with the waiting time at the practice. | ○ | | | ○ | ○ | | ○ | ○ |
|  | |  |  | |  |  | |  |
| My doctor is easily accessible for me (e.g., distance, transportation, accessibility, barrier-free access). | | ○ | ○ | | ○ | ○ | | ○ |
| My doctor and I can communicate without any language barriers (regarding the national language). | | ○ | ○ | | ○ | ○ | | ○ |
| My doctor has provided me with detailed information about endometriosis (e.g., etiology, disease progression, symptoms). | | ○ | ○ | | ○ | ○ | | ○ |
| My doctor has provided me with detailed information about different treatment options for endometriosis. | | ○ | ○ | | ○ | ○ | | ○ |
| My doctor has discussed the benefits of each treatment option with me. | | ○ | ○ | | ○ | ○ | | ○ |
| My doctor has discussed the disadvantages of each treatment option with me (e.g., side effects). | | ○ | ○ | | ○ | ○ | | ○ |
| My doctor has coordinated the therapy/therapies with me. | | ○ | ○ | | ○ | ○ | | ○ |
| My doctor considers my current needs in the treatment (e.g., desire to have children, hormone-free therapy). | | ○ | ○ | | ○ | ○ | | ○ |
| My doctor has informed me about psychological support options (e.g., therapy sessions) for coping with endometriosis | | ○ | ○ | | ○ | ○ | | ○ |

**[G08Q03] What do you wish for in endometriosis care and where do you see room for improvement?**

Please enter your answer here:

Open text

**[G08Q04] Is there anything else you would like to share?**

Please enter your answer here:

Open text

# Questions on demographics

Finally, we would like to ask you a few general questions about yourself.

**[G09Q00] What sex were you assigned at birth?**

Please choose one of the following answers:

- Female

- Male

**[G09Q01] Which gender do you identify with?**

Please choose one of the following answers:

- Female

- Male

- Non-binary

- Other (open text)

**[G09Q02] How old are you?**

Please enter your answer here (in years):

Open text

**[G09Q03] What is the postal code of your place of residence?**

Please enter your answer here:

Open text

**[G09Q04] How many inhabitants does your place of residence have?**

Please choose one of the following answers:

- Under 2,000

- Between 2,000 and 4,999

- Between 5,000 and 9,999

- Between 10,000 and 19,999

- Between 20,000 and 49,999

- Between 50,000 and 99,999

- Over 99,999

**[G09Q05] What type of health insurance do you have?**

Please choose one of the following answers:

- Statutory

- Private

- Not insured

**[G09Q06] Is German your native language?**

Please choose one of the following answers:

- Yes

- No

**[G09Q07] How would you rate your German language skills?** (Only displayed if “no” in [G09Q06])

Please choose one of the following answers:

- Very good

- Good

- Moderate

- Poor

- Very poor

**[G09Q09] In which language do you communicate with your treating physician?** (Only displayed if “no” in [G09Q06])

Please enter your answer here:

Open text

**[G09Q10] What is the highest general school qualification you have obtained?**

Please choose one of the following answers:

- I am still a student

- I left school without a qualification

- Lower secondary school certificate or equivalent

- Polytechnical Secondary School of the GDR with completion of 8th or 9th grade

- General Certificate of secondary school or equivalent

- Polytechnical Secondary School of the GDR with completion of 10th grade

- Technical college entrance qualification

- A-levels/ General university entrance qualification/

- Other (open text)

**[G09Q11] What is the highest vocational qualification you have obtained?**

Please choose one of the following answers:

- I am currently in training / studying

- I have no vocational qualification and am not in vocational training

- Apprenticeship (company-based vocational training)

- Vocational school qualification, e.g. from a vocational school or college

- Degree from a technical school, master technician school, academy of administration and business, or vocational academy

- Bachelor's degree, diploma from a technical college

- Master's degree, university diploma, Magister, state examination, doctorate

- Other (open text)

**[G09Q12] Which of the following best describes your employment situation?**

Select all answers that apply:

- Attending a general school

- Studying

- Vocational training

- Federal voluntary service, voluntary social or ecological year

- Care leave, maternity leave, parental leave

- Employed full-time

- Employed part-time (including partial retirement or midijob)

- Marginal or short-term employment (minijob, one-euro job, seasonal work)

- Retired/ pensioner

- Unemployed

- Unable to work

- Housewife/ househusband

- Other (open text)

**[G09Q13] Why are you unable to work?** (Only displayed if “unable to work” in [G09Q12])

Please choose one of the following answers:

- Due to endometriosis

- For other reasons

**[G09Q14] Have you had to reduce your working hours because of endometriosis?**(Only displayed if “Vocational training” or “Federal voluntary service, voluntary social or ecological year” or “On care leave, maternity leave, parental leave” or “Employed full-time” or “Employed part-time (including partial retirement or midi-job)” or “Minor or short-term employment (mini-job, one-euro job, seasonal work)“ or “retired/ pensioner” or “unemployed” or “housewife/ househusband” in [G09Q12])

Please choose one of the following answers:

- Yes

- No

**[G09Q15] How would you rate your general state of health?**

Please choose one of the following answers:

- Very good

- Good

- Moderate

- Poor

- Very poor

Thank you for participating in our survey.

If you have further comments, contact us at: endometriose-studie@gmx.de.
