# Supplementary material for: Patient satisfaction regarding medical care for endometriosis in Germany: an exploratory cross-sectional study
Source: BMC Womens Health. 2026 Mar 20;26:197. doi: 10.1186/s12905-026-04408-z (PMC13063557; doi:10.1186/s12905-026-04408-z)
Supplement: Supplementary file 3 — Supplementary Material 3. [file 12905_2026_4408_MOESM3_ESM.docx]

# Supplementary Material: Analysis of patient satisfaction by federal state

Fig. 1 shows patient satisfaction by federal state in Germany, revealing notable regional differences. Saxony had the highest satisfaction rate at 73.81% (SD = 16.33), although this finding is based on data from only six participants. Lower but still relatively high satisfaction levels were recorded in Lower Saxony (61.17%, SD = 14.78, n = 56) and Baden-Württemberg (60.83%, SD = 17.17, n = 38). Similarly, Bremen and North Rhine-Westphalia showed satisfaction rates above 60%, at 60.56% (SD = 14.56, n = 37) and 60.50% (SD = 15.29, n = 54), respectively. In contrast, the lowest satisfaction rates were observed in Thuringia (46.14%, SD = 18.40, n = 5), Hesse (49.25%, SD = 16.75, n= 18), and Brandenburg (51.12%, SD = 19.71, n = 7). No data was available for Mecklenburg-Vorpommern (Supplementary table 1).


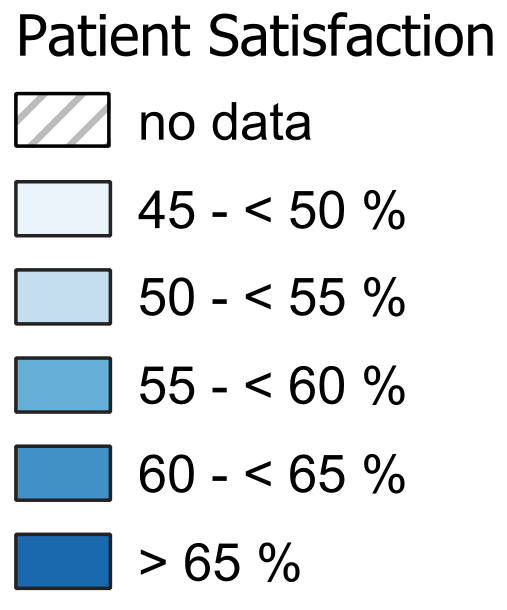

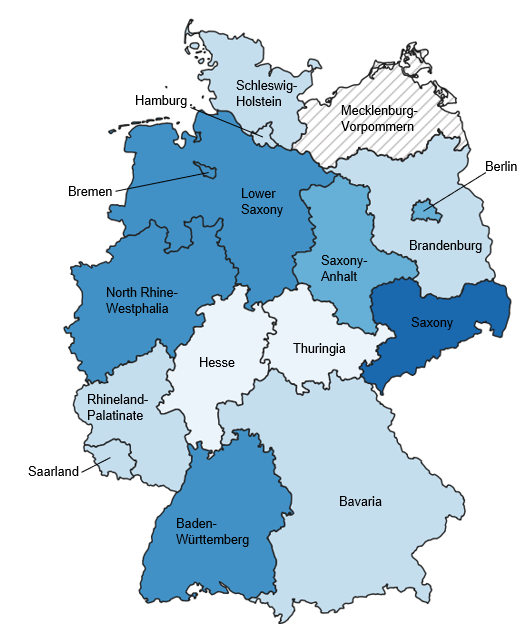


**Fig. 1** Patient satisfaction by federal state in Germany

**Supplementary Table 1** Patient satisfaction by federal state in Germany (N = 326)

| Federal state | N (%) | Mean satisfaction in % | SD |
| --- | --- | --- | --- |
| Saxony | 6 (1.84) | 73.81 | 16.33 |
| Lower Saxony | 56 (17.18) | 61.17 | 14.78 |
| Baden-Württemberg | 38 (11.66) | 60.83 | 17.17 |
| Bremen | 37 (11.35) | 60.56 | 14.56 |
| North Rhine-Westphalia | 54 (16.56) | 60.50 | 15.29 |
| Saxony-Anhalt | 5 (1.53) | 58.29 | 9.07 |
| Berlin | 14 (4.29) | 55.61 | 12.40 |
| Bavaria | 42 (12.88) | 54.32 | 15.20 |
| Saarland | 2 (0.61) | 54.29 | 13.13 |
| Rhineland-Palatinate | 11 (3.37) | 53.77 | 13.95 |
| Hamburg | 16 (4.91) | 52.72 | 21.59 |
| Schleswig-Holstein | 15 (4.60) | 52.62 | 16.80 |
| Brandenburg | 7 (2.15) | 51.12 | 19.71 |
| Hesse | 18 (5.52) | 49.25 | 16.75 |
| Thuringia | 5 (1.53) | 46.14 | 18.40 |
| Mecklenburg-Vorpommern | 0 (0.00) | Not available | Not available |

Satisfaction rates in this study on medical care of endometriosis revealed geographical variability, ranging from 46.14% in Thuringia to 73.81% in Saxony. These findings suggest potential regional variability in patient satisfaction with care provided by gynecologists and other specialists for endometriosis, which may reflect underlying differences in healthcare accessibility, resources, or other contextual factors. Previous research on general hospital care reported higher satisfaction levels among patients in the Eastern German federal states [1]. Although the present study focused on outpatient specialist care for endometriosis, the findings highlight the potential influence of regional factors on patient satisfaction and the need to address these disparities. It is important to note that the number of participants per federal state varied in this study, ranging from 2 respondents in Saarland to 56 respondents in Lower Saxony. Additionally, no data were available for Mecklenburg-Vorpommern. These discrepancies in sample sizes across regions may limit the generalizability of the findings and warrant caution when interpreting the observed regional differences in patient satisfaction.

**Reference**

1. von Wedel P, Hagist C, Liebe J-D, Esdar M, Hübner U, Pross C. Effects of hospital digitization on clinical outcomes and patient satisfaction: nationwide multiple regression analysis across German hospitals. Journal of medical Internet research. 2022;24(11):e40124.
