# Supplementary material for: Patient satisfaction regarding medical care for endometriosis in Germany: an exploratory cross-sectional study
Source: BMC Womens Health. 2026 Mar 20;26:197. doi: 10.1186/s12905-026-04408-z (PMC13063557; doi:10.1186/s12905-026-04408-z)
Supplement: Supplementary file 4 — Supplementary Material 4. [file 12905_2026_4408_MOESM4_ESM.docx]

# Supplementary Material

**Supplementary Table 1** Remaining descriptive statistics (N = 350)

| **Variables** | **Total** **n (%)** | **Confirmed diagnosis** **n (%)** | **Suspected diagnosis** **n (%)** | **Self-assumption** **n (%)** |
| --- | --- | --- | --- | --- |
| **N** | 350 (100.00) | 74 (78.29) | 61 (17.43) | 15 (4.29) |
| **Sex** |  |  |  |  |
| Female | 344 (100.00) | 269 (100.00) | 61 (100.00) | 14 (100.00) |
| Missing | 6 |  |  |  |
| **Population size** |  |  |  |  |
| Less than 2,000 | 34 (10.06) | 28 (10.53) | 4 (7.02) | 2 (13.33) |
| 2,000 - 4,999 | 26 (7.69) | 23 (8.65) | 1 (1.75) | 2 (13.33) |
| 5,000 - 9,999 | 27 (7.99) | 21 (7.89) | 5 (8.77) | 1 (6.66) |
| 10,000 - 19,999 | 52 (15.38) | 44 (16.54) | 6 (10.53) | 2 (13.33) |
| 20,000 - 49,999 | 60 (17.75) | 52 (19.55) | 7 (12.28) | 1 (6.66) |
| 50,000 - 99,999 | 27 (7.99) | 19 (7.14) | 6 (10.53) | 2 (13.33) |
| More than 99,999 | 112 (33.14) | 79 (29.70) | 28 (49.12) | 5 (33.33) |
| Missing | 12 |  |  |  |
| **Native language** |  |  |  |  |
| German | 333 (96.80) | 262 (97.76) | 56 (91.80) | 15 (100.00) |
| Other | 11 (3.20) | 6 (2.24) | 5 (8.20) | 0 (0.00) |
| Missing | 6 |  |  |  |
| **German language skills of non-native speakers*** |  |  |  |  |
| Very good | 10 (90.91) | 5 (83.33) | 5 (100.00) |  |
| Good | 0 (0.00) | 0 (0.00) | 0 (0.00) |  |
| Fair | 1 (9.09) | 1 (16.67) | 0 (0.00) |  |
| Poor | 0 (0.00) | 0 (0.00) | 0 (0.00) |  |
| Very poor | 0 (0.00) | 0 (0.00) | 0 (0.00) |  |
| Missing | 6 |  |  |  |
| **Incapacity to work** | 13 (3.77) | 12 (4.46) | 1 (1.64) | 0 (0.00) |
| Due to Endometriosis* | 9 | 8 | 0 |  |
| Missing | 5 |  |  |  |
| **Reduction of working hours due to  endometriosis*** | 104 (37.96) | 91 (41.18) | 12 (27.91) | 1 (10.00) |
| Missing | 9 |  |  |  |
| **General health status** |  |  |  |  |
| Very good | 22 (6.38) | 15 (5.58) | 5 (8.20) | 2 (13.33) |
| Good | 99 (28.70) | 69 (25.65) | 25 (40.98) | 5 (33.33) |
| Fair | 162 (46.96) | 132 (48.07) | 23 (37.70) | 7 (46.67) |
| Poor | 51 (14.78) | 44 (16.36) | 6 (9.84) | 1 (6.67) |
| Very poor | 11 (3.19) | 9 (3.35) | 2 (3.28) | 0 (0.00) |
| Missing | 5 |  |  |  |

Percentages are calculated from valid (non-missing) values, while the total N (N = 350) represents the complete sample.

*Due to filter questions, not all participants received the complete questionnaire.

Supplementary Table 2 summarizes the descriptive statistics for the individual questions on the assessment of patient satisfaction. The mean scores range from 1.88 to 4.04, with SDs between 0.99 and 1.36, indicating some variability in the responses. The minimum and maximum values reflect the range of responses.

**Supplementary Table 2** Descriptive statistics for individual questions on patient satisfaction (PSQ-18) (N = 350)

| Questions | Mean | SD | Min | Max |
| --- | --- | --- | --- | --- |
| Doctors are good about explaining the reason for medical tests | 3.37 | 1.23 | 1 | 5 |
| I think my doctor’s office has everything needed to provide complete medical care | 3.01 | 1.28 | 1 | 5 |
| The medical care I have been receiving is just about perfect | 2.25 | 1.12 | 1 | 5 |
| Sometimes doctors make me wonder if their diagnosis is correct | 3.17 | 1.32 | 1 | 5 |
| I feel confident that I can get the medical care I need without being set back financially | 2.10 | 1.16 | 1 | 5 |
| When I go for medical care, they are careful to check everything when treating and examining me | 3.46 | 1.24 | 1 | 5 |
| I have to pay for more of my medical care than I can afford | 2.87 | 1.29 | 1 | 5 |
| I have easy access to the medical specialists I need | 2.12 | 1.18 | 1 | 5 |
| Where I get medical care, people have to wait too long for emergency treatment | 2.86 | 1.24 | 1 | 5 |
| Doctors act too businesslike and impersonal towards me | 3.47 | 1.26 | 1 | 5 |
| My doctors treat me in a very friendly and courteous manner | 4.04 | 0.99 | 1 | 5 |
| Those who provide my medical care sometimes hurry too much when they treat me | 2.88 | 1.32 | 1 | 5 |
| Doctors sometimes ignore what I tell them | 2.97 | 1.33 | 1 | 5 |
| I have some doubts about the ability of the doctors who treat me | 3.14 | 1.36 | 1 | 5 |
| Doctors usually spend plenty of time with me | 3.21 | 1.20 | 1 | 5 |
| I find it hard to get an appointment for medical care right away | 2.55 | 1.28 | 1 | 5 |
| I am dissatisfied with some things about the medical care I receive | 1.88 | 1.00 | 1 | 5 |
| I am able to get medical care whenever I need it | 2.65 | 1.15 | 1 | 5 |

**Supplementary Table 3** Descriptive statistics for each patient satisfaction subscale
(N = 350)

|  | PSQ-18 | | | PSQ-18+ | | |
| --- | --- | --- | --- | --- | --- | --- |
| Domains | Mean | SD | Satisfaction in % | Mean | SD | Satisfaction in % |
| General Satisfaction | 2.07 | 0.93 | 41.34 | 2.07 | 0.93 | 41.34 |
| Technical Quality | 3.19 | 1.03 | 63.87 | 3.24 | 0.93 | 64.75 |
| Interpersonal Manner | 3.76 | 1.03 | 75.14 | 3.74 | 0.87 | 74.78 |
| Communication | 3.17 | 1.12 | 63.40 | 3.62 | 0.80 | 72.44 |
| Financial Aspects | 2.48 | 1.10 | 49.69 | 2.48 | 1.10 | 49.69 |
| Time Spent with Doctor | 3.05 | 1.17 | 60.91 | 3.05 | 1.17 | 60.91 |
| Accessibility and Convenience | 2.54 | 0.91 | 50.87 | 2.79 | 0.80 | 55.82 |
| Patient Counseling |  |  |  | 2.28 | 1.05 | 45.52 |
| Shared Decision-Making |  |  |  | 3.20 | 1.09 | 63.96 |
| Overall Satisfaction | **2.89** | **0.80** | **57.89** | **2.94** | **0.73** | **58.80** |

**Supplementary Table 4** Univariate linear regression analyses for sociodemographics (PSQ-18 and PSQ-18+)

|  |  | **PSQ-18** | | | **PSQ-18+** | | |
| --- | --- | --- | --- | --- | --- | --- | --- |
| **Predictors** | N | Intercept | β (CI) | p | Intercept | β (CI) | p |
| **Age** | 345 | 56.2 | 0.06 (-0.23; 0.35) | 0.5867 | 55.6 | 0.11 (-0.16; 0.37) | 0.3023 |
| **ISCED** | 344 | 58.1 |  |  | 58.8 |  |  |
| Low |  |  | 0.21 (-15.80; 16.21) | 0.9730 |  | -1.70 (-16.39; 12.99) | 0.7646 |
| Middle |  |  | -0.16 (-4.68; 4.36) | 0.9262 |  | 0.22 (-3.93; 4.37) | 0.8909 |
| High |  |  | Reference |  |  | Reference |  |
| **Health insurance** | 345 | 57.2 |  |  | 58.3 |  |  |
| Private |  |  | 8.61 (0.92; 16.31) | 0.0040 |  | 6.64 (-0.45; 13.73) | 0.0158 |
| Statutory |  |  | Reference |  |  | Reference |  |
| **Population size** | 338 | 57.4 |  |  | 58.8 |  |  |
| Less than 2,000 |  |  | 1.41 (-6.73; 9.54) | 0.6558 |  | -0.12 (-7.59; 7.35) | 0.9669 |
| 2,000 - 4,999 |  |  | 0.35 (-8.70; 9.39) | 0.9213 |  | 0.14 (-8.16; 8.44) | 0.9649 |
| 5,000 - 9,999 |  |  | 0.19 (-8.71; 9.10) | 0.9554 |  | -0.41 (-8.58; 7.77) | 0.8982 |
| 10,000 - 19,999 |  |  | 2.12 (-4.85; 9.09) | 0.4342 |  | 0.97 (-5.43; 7.37) | 0.6965 |
| 20,000 - 49,999 |  |  | -0.44 (-7.09; 6.20) | 0.8635 |  | -1.00 (-7.10; 5.10) | 0.6741 |
| 50,000 - 99,999 |  |  | 3.55 (-5.35; 12.46) | 0.3049 |  | 2.33 (-5.84; 10.51) | 0.4632 |
| More than 99,999 |  |  | Reference |  |  | Reference |  |
| **Degree of  urbanization** | 316 | 55.9 |  |  | 59.2 |  |  |
| Rural area |  |  | -1.89 (-7.95; 4.16) | 0.4812 |  | -2.13 (-7.70; 3.44) | 0.3234 |
| Town |  |  | 0.73 (-4.70; 6.16) | 0.7275 |  | -0.05 (-5.04; 4.94) | 0.9775 |
| City |  |  | Reference |  |  | Reference |  |

Results of the univariate and multiple linear regression predicting patient satisfaction for PSQ-18+. Unstandardized Regression coefficients (β), and 95% confidence interval Bonferroni corrected (CI), Significance level: p < 0.01.

**Supplementary Table 5** Univariate and Multiple linear regression analyses for disease
severity and diagnostic delay (PSQ-18+)

|  |  | **Univariate Regression** | | | **Multiple Regression** | | |
| --- | --- | --- | --- | --- | --- | --- | --- |
| **Predictors** | N | Intercept | β (CI) | p | Intercept | β (CI) | p |
| Number of symptomatic days per month ^a, f, h, j^ | 325 | 61.4 | -0.16 (-0.39; 0.07) | 0.0562 | 59.7 | -0.02 (-0.30; 0.26) | 0.8431 |
| Number of days restricted in daily life per month ^a, f, h, j^ | 325 | 61.7 | -0.24 (-0.49; 0.00) | 0.0063 | 58.3 | -0.13 (-0.43; 0.17) | 0.2336 |
| Number of days on sick leave per month ^a, f, h, j^ | 318 | 59.8 | -0.17 (-0.52; 0.19) | 0.1857 | 60.4 | 0.01 (-0.40; 0.42) | 0.9591 |
| Clinical diagnosis time (confirmed) ^b, d, e, g, i, k^ | 235 | 60.8 | -0.32 (-0.76; 0.13) | 0.0482 | 61.1 | -0.20 (-0.74; 0.35) | 0.3149 |
| Clinical diagnosis time (suspected) ^b, d, e, g, i, k^ | 47 | 60.2 | -0.99 (-2.24; 0.27) | 0.0266 | 41.0 | -0.61 (-1.93; 0.71) | 0.1839 |
| Overall diagnosis time (confirmed) ^c, d, e, g, i, k^ | 228 | 59.6 | -0.07 (-0.50; 0.35) | 0.6332 | 66.2 | -0.08 (-0.59; 0.44) | 0.6866 |
| Overall diagnosis time (suspected) ^c, d, e, g, i, k^ | 41 | 62.4 | -0.91 (-2.03; 0.21) | 0.0217 | 48.2 | -0.39 (-1.76; 0.98) | 0.4049 |
| Number of initial symptoms ^a, k^ | 309 | 63.1 | -0.32 (-0.67; 0.03) | 0.0114 | 63.3 | -0.32 (-0.69; 0.05) | 0.0157 |
| Number of symptoms ^a, h, j^ * | 334 | 8.1 | -0.03 (-0.05; -0.01) | <0.0001 | 7.9 | -0.03 (-0.05; -0.01) | 0.0002 |

Results of the univariate and multiple linear regression predicting patient satisfaction for PSQ-18+. Unstandardized Regression coefficients (β), and 95% confidence interval Bonferroni corrected (CI), Significance level univariate: p < 0.00625, Significance level multiple: p < 0.00556.

Predictors were adjusted for the confounders: ^a^ age, ^b^ age at first consultation with a doctor, ^c^ age at onset of symptoms, ^d^ degree of urbanization, ^e^ type of health insurance, ^f^ general health status, ^g^ ISCED, ^h^ number of chronic diseases, ^i^ number of initial symptoms, ^j^ number of therapies, ^k^ prescription of the birth control pill prior to diagnosis.

*Square-root transformation of outcome variable due to violation of normal distribution assumption.
